# Echocardiographic Features of Cardiac Involvement in Myotonic Dystrophy 1: Prevalence and Prognostic Value

**DOI:** 10.3390/jcm12051947

**Published:** 2023-03-01

**Authors:** Vincenzo Russo, Antonio Capolongo, Roberta Bottino, Andreina Carbone, Alberto Palladino, Biagio Liccardo, Gerardo Nigro, Michał Marchel, Paolo Golino, Antonello D’Andrea

**Affiliations:** 1 Cardiology Unit, Department of Medical Translational Sciences, University of Campania “Luigi Vanvitelli”, Monaldi Hospital, 80121 Naples, Italy; 2 Cardiomyology and Genetic Section, University of Campania “Luigi Vanvitelli”, 80138 Naples, Italy; 3 1st Department of Cardiology, Medical University of Warsaw, 02-091 Warsaw, Poland; 4 Department of Cardiology, Umberto I Hospital, 84014 Nocera Inferiore, Italy

**Keywords:** myotonic dystrophy type 1, echocardiography, left ventricular dysfunction, global longitudinal strain, arrhythmias, sudden cardiac death

## Abstract

Myotonic dystrophy type 1 (DM1) is the most common muscular dystrophy in adults. Cardiac involvement is reported in 80% of cases and includes conduction disturbances, arrhythmias, subclinical diastolic and systolic dysfunction in the early stage of the disease; in contrast, severe ventricular systolic dysfunction occurs in the late stage of the disease. Echocardiography is recommended at the time of diagnosis with periodic revaluation in DM1 patients, regardless of the presence or absence of symptoms. Data regarding the echocardiographic findings in DM1 patients are few and conflicting. This narrative review aimed to describe the echocardiographic features of DM1 patients and their prognostic role as predictors of cardiac arrhythmias and sudden death.

## 1. Introduction

Myotonic dystrophy type 1 (DM1) is the most common inherited multisystem neuromuscular disease, caused by an unstable expansion of a trinucleotide (CTG) repeat on chromosome 19 in the 3′ untranslated region of the myotonic dystrophy protein kinase gene [1]. The estimated prevalence is 1:8000 [2] adults. The geographic and ethnic distribution is very uneven among different populations. DM1 is inherited with an autosomal dominant transmission, incomplete penetrance, and variable expressivity. The pathophysiological mechanisms of DM1 are mainly based on ribonucleic acid toxicity. CTG repeats are transcribed into ribonucleic acid but not translated [3]; the accumulation of ribonucleic acid causes myocyte hypertrophy, fatty infiltration, interstitial fibrosis and myofibrillar degeneration [4,5]. DM1 is characterized by muscle weakness, myotonia, cataracts, cardiac, respiratory and endocrine disturbances, excessive daytime sleepiness, cognitive and personality trait abnormalities and skin alterations. In addition, insulin resistance, increased waist circumference, dyslipidemia and reduced levels of adiponectin are common. DM1 is also associated with an increased risk of developing several types of benign or malignant tumors. No curative or disease-modifying treatments are currently available, and the management focuses on genetic counseling, preserving function and independence, preventing cardiopulmonary complications, including those related to life-threatening arrhythmias, and symptomatic treatments.

Cardiac involvement is reported in about 80% of cases [2] and often precedes muscular impairment. The DM1 cardiac phenotype is broad and includes conduction disturbances, arrhythmias, subclinical diastolic and systolic dysfunction in the early stage of disease [6,7,8,9]; in contrast, severe ventricular systolic dysfunction occurs in the late stage of disease. In fact, dilated cardiomyopathy and end-stage cardiomyopathy are uncommon [6,7]. Myocardial infarction is responsible for about 5% of the cardiovascular causes of death in DM1 patients. Sudden cardiac death occurs in 30% of DM1 patients [10,11]. 

Data regarding the echocardiographic findings in DM1 patients are few and conflicting [12,13,14,15,16,17,18,19]. The aim of this narrative review is to describe the echocardiographic features of DM1 patients and their prognostic role as predictors of cardiac arrhythmias and sudden death.

### 1.1. Left Ventricular Systolic Dysfunction

The prevalence of left ventricular systolic dysfunction, defined as left ventricular ejection fraction (LVEF) <55%, is 13.8% in DM1 patients, about 4.5-fold higher than in the general population [20,21]. The onset of heart failure symptoms occurs at the median age of 47 years [20]. DM1 patients with LVEF <55% are more likely male and older, and show a higher prevalence of conduction disorders and atrial arrhythmias; conversely, no association between LVEF and disease duration, number of CTG repeats or neuromuscular disability has been shown [22]. Left ventricular systolic dysfunction is a prognostic factor of pivotal importance in DM1 patients since it was associated with a significantly increased risk of overall mortality, sudden death and ventricular arrhythmias [23].

Although trials showing benefits from the treatment of heart failure in DM1 are lacking, it seems reasonable that treatment should be started early. In particular, the administration of angiotensin-converting enzyme (ACE) inhibitors and angiotensin II receptor antagonists (ARB) could be of particular benefit in DM1 due to anti-fibrotic properties [7] and they are recommended when LVEF is lower than 50%. The use of beta-blockers should be reserved for patients without AV conduction defects or recipients of pacemakers and/or cardioverter defibrillators; dosage should be titrated according to the individual response and toleration. According to international guidelines, DM1 patients with LVEF ≤ 35%, despite guideline-directed medical therapy, have a class I indication to receive ICD therapy, if concordant with the patient’s goals of care and clinical status [24]. Cardiac resynchronization therapy is indicated in DM1 patients with LVEF ≤35%, sinus rhythm, left bundle branch block with QRS duration ≥150 ms, and NYHA class II to class IV symptoms, or suspected right ventricular pacing–induced cardiomyopathy despite guideline-directed medical therapy [24].

### 1.2. Left Ventricular Strain

Left ventricular deformation imaging by tissue Doppler imaging (TDI) and speckle-tracking echocardiography (STE) has emerged as a robust means for assessing the left ventricular function that has notable advantages over LVEF measured by two-dimensional (2D) echocardiography. Strain measures the lengthening, shortening, thickening and rotational capacity of the myocardium. Although LVEF may be impacted by various diseases, strain analysis may allow for a deeper understanding of disease states.

Subclinical systolic dysfunction evaluated through left ventricular strain has been reported with a prevalence of up to 28% [25].

According to Wahbi et al. [26], the speckle tracking global longitudinal strain (GLS) analysis detected early asymptomatic contractility abnormalities in DM1 patients with normal LVEF. In particular, DM1 patients showed a lower apical four chambers GLS compared to controls (−17.8 ± 2.5 vs. −19.2 ± 2.3; *p* = 0.01), which was significantly correlated with PR interval. The increased circumferential contractility component (−19.5 ± 3.5 vs. −17.8 ± 2.4; *p* = 0.01) might constitute a mechanism to compensate for the longitudinal abnormalities. These results suggested the existence of a link between conduction disturbance and subclinical or overt myocardial function abnormalities in DM1 patients [26].

Preliminary findings by Sousa et al. [27] showed that GLS (−16.6 ± 3.6% vs. −18.7 ± 1.8%, *p* = 0.022) was lower in DM1 patients than in healthy volunteer controls and no differences regarding segmental longitudinal deformities were found. In addition, the presence of functional disability was associated with lower GLS. Finally, a positive correlation between GLS impairment and PR interval duration was shown; patients with lower GLS had delayed atrioventricular conduction.

In a large single-center study, including 129 DM1 patients, by Petry et al. [28], 21.7% of the study population had abnormal GLS, above −15.9%, and among them, 60% had preserved LVEF > 50% [28]. There was a trend towards a positive correlation between GLS and PR interval.

In a prospective single-center study including 33 DM1 patients with preserved LVEF > 55%, Garcia et al. [29] showed an early deformation impairment in both radial (20.0 ± 9.8 vs. 27.5 ± 14.9; *p* = 0.02) and longitudinal strain (−18.0 ± 1.9 vs. −19.1 ± 2.4; *p* = 0.03) compared to controls; the regional analysis showed a marked GLS alteration at the apex (−20.0 ± 3.3 vs. −22.7 ± 3.1; *p* < 0.001).

GLS impairment was a strong predictor of a composite of cardiovascular events (all-cause mortality, type 2 Mobitz 2 and type 3 atrioventricular block, symptomatic sino-atrial block, HV interval ≥ 70 ms at invasive electrophysiology exploration, LVEF ≤ 45% and newly developed atrial fibrillation) in asymptomatic DM1 patients. In particular, a GLS cut-off value of −17.2% showed a sensitivity of 93% and specificity of 72% to predict events. DM1 patients with GLS ≥ −17.2% have a 1.4-fold increased risk of cardiovascular events compared with patients with GLS < −17.2% [13].

### 1.3. Left Ventricular Diastolic Dysfunction

The prevalence of left ventricular diastolic dysfunction in DM1 patients ranged from 24% to 29% [14,15]. It has been proposed that the myocardial degenerative process as well as poor relaxation due to myotonia may contribute to the diastolic dysfunction [30] and may explain this high prevalence.

The most common echocardiographic features of diastolic dysfunction were the increased peak of mitral E velocity, the prolonged isovolumic relaxation time, and the reduced deceleration time of the early filling E wave velocity [15,16]. In contrast, no differences were found in mitral E/A ratio, the lateral and septal peak of early diastolic velocity (e’) at tissue Doppler imaging, compared to healthy subjects [16].

The myocardial performance index (MPI) is a Doppler-derived index that reflects the global left ventricular function, regardless of the contribution of systolic or diastolic phases [19,31]. The MPI is impaired in patients with DM1 [31]. Despite their limited use, color-Doppler myocardial imaging (CDMI) and integrated backscatter (IBS) can distinguish DM1 patients with systolic or diastolic dysfunction, and the intensity of IBS is dependent on the degree of myocardial fibrosis [32].

Cori et al. observed that the IBS amplitude of the interventricular septum and LV posterior wall was increased in DM1 patients [31]. The echocardiographic signs of diastolic dysfunction can be present even in DM1 patients with no symptoms of heart failure or left ventricular systolic dysfunction [15]. Differently from systolic dysfunction, diastolic dysfunction does not appear to be associated with either electrocardiographic abnormalities or age among DM1 patients [33].

### 1.4. Right Ventricular Function

The right ventricular function in DM1 patients has been poorly investigated through echocardiography. In two case-control studies [17,34], including a total of 51 DM1 patients, a significant prolongation of isovolumic contraction time (IVCT), isovolumic relaxation time (IVRT) and RV Tei index (IVCT + IVRT/ejection time) compared to controls was observed. Moreover, a shorter ejection time; a higher right ventricular–right atrial pressure drop; a reduction in RV-free wall Sm and Am velocities; and a reduction in RV-free wall systolic strain have been shown. The RV-IVCT was correlated with PR interval (r = 0.64, *p* < 0.001) [17].

### 1.5. Left Atrial Function

The principal role of the left atrium is to modulate left ventricular filling and cardiovascular performance by functioning as a reservoir for pulmonary venous return during ventricular systole, a conduit for pulmonary venous return during early ventricular diastole, and a booster pump that augments ventricular filling during late ventricular diastole. The left atrial deformation via echocardiographic strain and strain rate allows for accurate and reproducible analysis of left atrial function [35,36]. An abnormal left atrial strain may more accurately estimate cardiac filling pressures and predict functional status, peak VO2, and mortality when compared with conventional noninvasive assessments [37,38].

In an observational study by Guedes et al. [36], DM1 patients showed a significantly decreased left atrial (LA) longitudinal strain compared to healthy controls (22.85 ± 5.06 vs. 26.82 ± 5.15; *p* = 0.008), despite the fact that the two-dimensional echocardiography did not identify differences between the groups in left cardiac chamber size or parameters of diastolic function. These results may reflect a subclinical and early marker of atrial myocardial dysfunction in these patients. Moreover, the significant inverse relationship between LA longitudinal strain and age suggests a more rapid decline of left atrial function in DM1 patients. A possible relationship between the segmental pattern of myocardial deformation, detected by speckle tracking, and electrophysiological abnormalities at invasive evaluation has been hypotized [39].

In a case-control study including 50 DM1 patients, Russo et al. showed that the electrocardiographic indices of atrial electromechanical delay (inter-AEMD and intraleft-AEMD) were significantly increased in DM1 patients when compared with age and sex-matched healthy controls.

Atrial electromechanical delay duration is the sum of impulse propagation from the sinus node to the atria and atrial electromechanical coupling duration. The AEMD measurement was obtained by placing TDI sample volume on the lateral mitral annulus (named lateral PA), septal mitral annulus (septal PA) and right ventricular tricuspid annulus (RV PA). Time intervals from the onset of P-wave on surface-ECG to the beginning of A-wave (PA) representing atrial-electromechanical delay were obtained from the lateral mitral annulus, septal mitral annulus and right ventricular (RV) tricuspid annulus. The difference between septal PA and RV PA was defined as intra-right atrial AEMD; the difference between lateral PA and septal PA was defined as intra-left atrial AEMD; and the difference between lateral PA and RV PA was defined as inter-atrial AEMD. Previous studies evaluated the predictive role of intra-left atrial electromechanical delay for paroxysmal atrial fibrillation recurrence in some clinical conditions [40,41,42].

In the DM1 subgroup that showed paroxysmal atrial fibrillation during 30-day external loop monitoring, the inter-AEMD and the intraleft AEMD were significantly higher than in the DM1 subgroup without AF. A cut-off value of 39.2 milliseconds for intraleft AEMD had a sensitivity and specificity of 90% in identifying AF high risk DM1 patients. A cut-off value of 57.7 milliseconds for inter-AEMD had a sensitivity of 84.2% and a specificity of 93.5% in identifying DM1 patients at high risk to develop AF [43,44,45].

Considering the high supraventricular arrhythmia risk and its consequences, the early identification of DM1-patients at high risk for AF is of pivotal importance for the optimization of clinical follow-up and medical therapy. Intra-left and inter-AEMD represent non-invasive, inexpensive, useful and simple parameters to assess the AF risk in DM1 patients.

**Figure 1:**
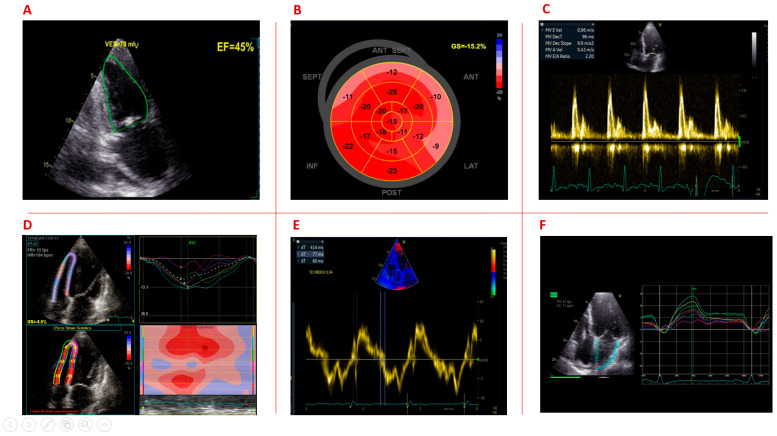
Overview of the echocardiographic functional abnormalities in DM1 patients. (**A**) Reduced left ventricular ejection fraction; (**B**) impaired left ventricular global longitudinal strain; (**C**) diastolic dysfunction; (**D**) impaired right ventricular longitudinal strain; (**E**) increased atrial electromechanical delay; (**F**) impaired left atrial strain.

Figure 1 summarizes the most common echocardiographic functional abnormalities in DM1 patients.

### 1.6. Structural Cardiac Abnormalities

Some structural cardiac abnormalities have been described across different studies [22,23,33,46,47,48]. Left ventricular (LV) hypertrophy, defined as the interventricular septal or LV posterior wall thickness of at least 11 mm, has a prevalence ranging from 19% to 29% [47,48]; LV dilatation, diagnosed when the LV end-diastolic diameter is higher than 51 mm, has a prevalence of 18.6% [48]. A significantly lower LV mass and higher prevalence of LV non-compaction were found in DM1 patients with normal systolic function compared to controls [46]. Left atrial dilatation, defined as LA diameter higher than 40 mm, was shown in 6.3% [48] of DM1 patients. Mitral valve prolapse is the most prevalent valvular heart disease and it was reported in 13.7–37% of DM1 patients [23,33,47,48]; in contrast, the prevalence of mild aortic regurgitation was lower than in the healthy control population [33]. The different prevalence of aortic regurgitation could be explained because hypertension is more common in the general population than in DM1 patients, due to the smooth muscle impairment of the vessels in DM1 [49].

**Figure 2:**
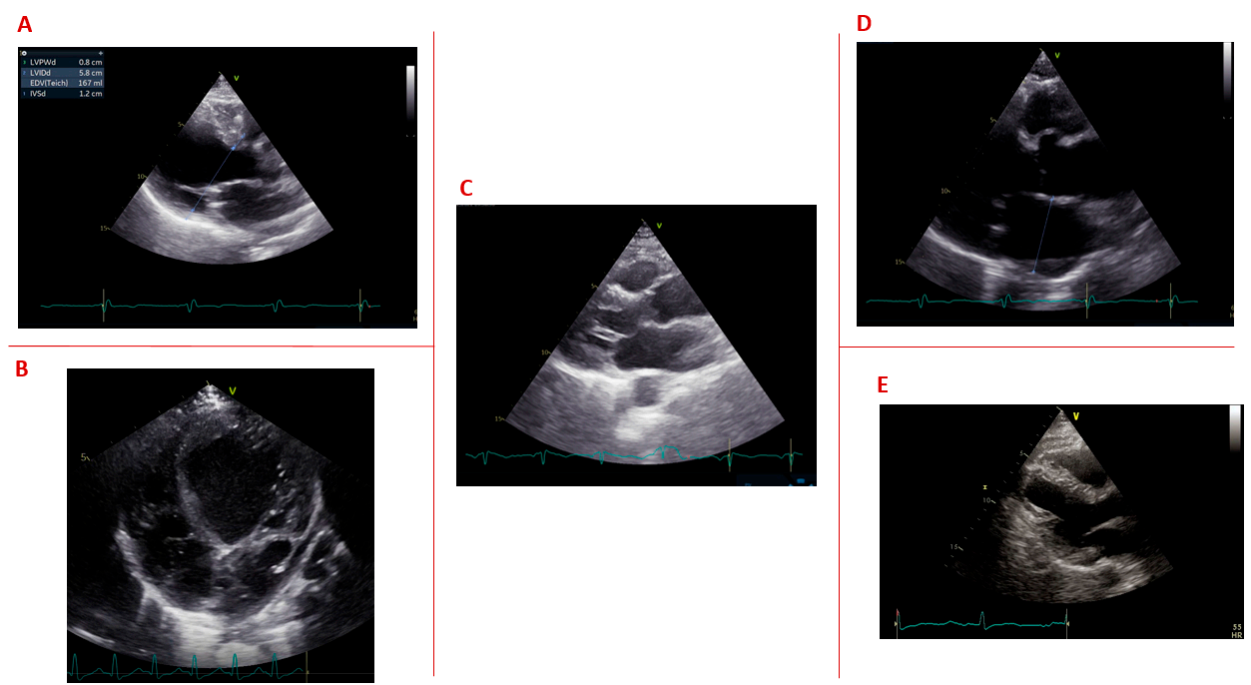
Overview of the echocardiographic structural abnormalities in DM1 patients. (**A**) Left ventricular dilatation; (**B**) myocardial non-compaction; (**C**) mitral valve prolapse; (**D**) left atrial enlargement; (**E**) left ventricular hypertrophy.

No clinical or genetic predictors of structural cardiac abnormalities were found [48]. The prognostic role of asymptomatic structural cardiac abnormalities has not been evaluated in DM1 patients. Finally, concerning structural abnormalities of the right ventricle, some studies described right ventricular dilatation [22], while other studies, such as the case-control study by Choudhary et al., showed no differences in end-systolic and end-diastolic right ventricular volumes, as well as in right atrial volume [46]. Figure 2 summarizes the most common echocardiographic structural abnormalities in DM1 patients.

### 1.7. Relation between Echocardiographic Features and Arrhythmias

Conduction system disease is the most prevalent cardiac abnormality in DM1 patients. The first-degree atrioventricular block is reported in 28.2–34.1% and QRS complex> 120 ms in 18.4–19.9% [28]. These electrocardiographic conduction abnormalities are independent predictors for a prolonged His-ventricle (HV) interval ≥ 70 ms on the electrophysiological study (EPS) [50], which early identifies a subgroup of DM1 patients in need of cardiac pacing [51]. Atrial fibrillation (AF), often asymptomatic, frequently occurs in DM1 patients with a prevalence of 11%, about 70-fold higher than the general population [9,52]; however, it could be even higher, about 25%, if we consider cardiac implanted electronic device detected AF events [53]. DM1 patients affected by AF were more often males, had lower left ventricular ejection fraction, electro-mechanical echocardiographic and electrocardiographic abnormalities [8]. AF has been associated with higher overall mortality in DM1 patients [9]. The prevalence rates of non-sustained and sustained ventricular tachycardia were 2.2% and 0.8%, respectively [54]. The personal history of non-sustained VT has been recently identified as the only independent predictor of sustained VT [55].

Several studies evaluated the bidirectional relationship between echocardiographic abnormalities and conduction disorders in DM1 patients [13,23,31,33,48,56]. If, on the one hand, the LVEF < 50% was associated with the risk of cardiac arrhythmias and conduction defects, such as atrioventricular block [56], then, on the other hand, PR interval ≥ 240 milliseconds and QRS duration ≥ 120 milliseconds were associated with the reduced LVEF. In such cases, the early onset of the left ventricular systolic dysfunction with symptomatic heart failure and left bundle branch block has been treated by biventricular pacing with a rapid resolution, suggesting that the early onset of heart failure could be related to the electromechanical delay caused by both intra- and inter-ventricular asynchrony. His delay leads to regional molecular changes in a non-coordinate contracting myocardium and accelerates the progression of the heart failure [57,58].

The intensity of the septal IBS echo correlates with the duration of the PR interval, thus suggesting a role of this echo parameter as a marker of conduction system alterations [31]. Finally, PR > 200 ms correlates with wall motion abnormalities, and QRS > 120 ms correlates with both regional wall motion abnormalities and left atrial dilatation [48].

**Figure 3:**
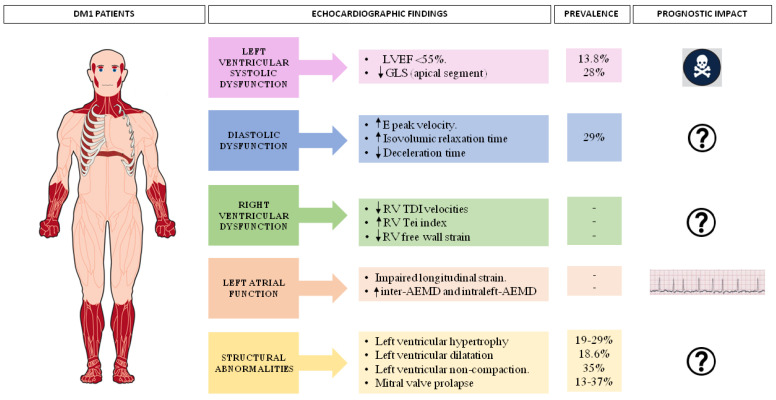
Overview of the prevalence and prognostic impact of echocardiographic structural abnormalities in DM1 patients.

Figure 3 summarizes the prevalence and the prognostic impact of echocardiographic functional abnormalities in DM1 patients.

### 1.8. Recommendations for Echocardiography in DM1 Patients

According to “Management of Cardiac Involvement Associated With Neuromuscular Diseases: A Scientific Statement From the American Heart Association”, an echocardiogram should be performed at the time of DM diagnosis, regardless of symptoms (Class I; Level of Evidence C). DM patients with palpitations, dizziness, syncope, non–sinus rhythm, PR interval > 240 ms, QRS duration > 120 ms or second- or third-degree atrioventricular block should be evaluated at least annually. DM patients with normal LVEF who lack the above characteristics should be reassessed by an echocardiogram every 2 to 4 years (Class IIa; Level of Evidence B) [59].

The more recent 2022 HRS expert consensus statement on the evaluation and management of arrhythmic risk in neuromuscular disorders [24] suggests that a comprehensive cardiac evaluation including physical examination, electrocardiogram (ECG), ambulatory ECG and cardiac imaging (echocardiography or cardiac magnetic resonance) at diagnosis with periodic retesting, once yearly, is recommended even in the absence of cardiac symptoms (Class I level of evidence B-NR).

### 1.9. Pharmacological and Device-Based Therapy

The assumption that cardiac dysfunction can be prevented (or at least attenuated) in DM1 patients has led to the belief that angiotensin converting enzyme-inhibithors (ACE-I) should be initiated at an early stage of the disease, rather than delayed until ventricular dilatation or systolic dysfunction become apparent. This clinical strategy is based on some phenotypic similarities between patients with DM1 and other cardiomyopathy associated with muscular dystrophy; however, no prospective studies have investigated this approach.

Of course, in DM1 patients with heart failure and reduced ejection fraction, the use of ACE-I/angiotensin-receptor blocker (ARB), angiotensin receptor-neprilysin inhibitors, mineralocorticoid receptor antagonists, beta-blockers and sodium-glucose co-transporter 2 are recommended based on clinical trials showing benefits in the absence of neuromuscular disorders (NMDs) [60]. However, it should be noted that the uptitration of these medications may be limited by the constitutional hypotension of DM1 patients [61].

Permanent cardiac pacing is indicated in patients with any second- and third-degree atrioventricular block or His-ventricle (HV) interval > 70 ms, regardless of the symptoms, and it may be considered in those with QRS > 120 ms and PR > 240 ms.

ICD implantation may be considered for all DM1 with permanent pacing indication and spontaneous or inducible ventricular arrhythmias, even when asymptomatic or with preserved cardiac function. Cardiac resynchronization therapy may be an option for DM1 patients with bundle branch block (especially left bundle branch block), who need permanent pacemaker implantation; however, there are currently only a few case reports about CRT therapy in DM1 patients) [57,58,62,63].

## 2. Future Perspectives

DM1 patients have a three-fold higher risk of sudden cardiac death (SCD) than age-matched healthy controls. The annual incidence of sudden death has been estimated at 0.53–1.16% [64,65]. SCD accounts for up to 33% of all deaths in DM1. Eve71.n if the mechanisms leading to SD remain controversial, the complete atrioventricular block, asystole and ventricular tachyarrhythmias may represent the most prevalent causes of SCD in DM1 patients. Independent predictors of sudden cardiac death are atrial tachyarrhythmias, PR interval ≥ 240 ms, QRS duration ≥ 120 ms, in particular left bundle branch block, second- or third-degree atrioventricular block, age, family history of sudden death, non-sustained VT and structural abnormalities in cardiac magnetic resonance (CMR) [66].

The emerging role of serum-specific biomarkers, such as copeptin or microRNAs, in predicting arrhythmias [67,68] and the ability of CMR in stratifying cardiac involvement have recently been hypothesized [69].

Since the early abnormalities of LV longitudinal deformation, in particular at apex, have been correlated with sudden cardiac death, prospective studies are needed to include these parameters among additional arrhythmic risk factors.

## 3. Conclusions

The prevalence of echocardiographic signs of systolic and diastolic dysfunction is high in DM1 patients. The reduced left ventricular ejection fraction is associated with an increased risk of overall mortality, sudden death and ventricular arrhythmias. The subclinical systolic dysfunction, assessed by GLS, is associated with a composite of cardiovascular events in asymptomatic DM1 patients. The apical GLS impairment seems to be the earlier and stronger predictor of a worse prognosis, including sudden cardiac death. The increased intra-left and inter-atrial electromechanical delays are correlated with a high risk to develop AF. The cardiologists involved in the management of DM1 patients should be aware of the preclinical impairment echocardiographic signs to identify DM1 patients in need of more intensive therapeutic management earlier (Table 1).

## Figures and Tables

**Table 1:** Main clinical studies on echocardiographic features in DM1 patients.

Study	Year	Study Design	DM1 Study Population (n)	DM1 Patients Age (Years)	Healthy Controls (n)	Echocardiographic Feature	Main Findings
**Left Ventricular Systolic Dysfunction**							
Russo [20]	2020	Systematic review	876	42.68	-	LVEF < 55%	The prevalence of LVEF < 55% was 13.8%.
Garcia [29]	2017	Prospective study	33	38.2 ± 12.9	33	LV GLS	DM1 patients exhibited significantly altered LV GLS, particularly at the apex (−20.0 ± 3.3 vs. −22.7 ± 3.1; *p* < 0.001), as compared with controls.
Garcia [13]	2017	Prospective study	46	40 [29–49]	-	LV GLS	LV GLS (cut-off value of −17.2%) predict cardiovascular events, regardless LVEF.
Guedes [36]	2017	Observational study	25	36.9 ± 16.0	25	LA and LV GLS	LA longitudinal strain is significantly decreased in patients with DM1 compared to controls (22.85 ± 5.06 vs. 26.82 ± 5.15 *p* = 0.008).
Petri [28]	2014	Cross-sectional study	129	44 (15)	-	GLS	The prevalence of abnormal GLS was 21.7% Abnormal GLS was above −15.9%; 60% had preserved LVEF> 50%.
Sousa [27]	2013	Case–control study	25	36.7 ± 12.5	13	GLS	DM1 patients showed a lower GLS than controls (−16.6 ± 3.6% vs. −18.7 ± 1.8%, *p* = 0.022). GLS correlates with PR interval duration.
**Right Ventricular Function**							
Lindqvist [46]	2010	Case–control study	36	45 ± 10	16	Right ventricular function by Doppler and RV strain	DM1 patients showed a prolonged IVCT and IRVT (both *p* < 0.05); shorter ET (*p* < 0.05); a higher right ventricular–right atrial pressure drop (23 ± 7 vs. 18 ± 2 mm Hg, *p* < 0.05); a reduction in RV free wall Sm (*p* < 0.001) and Am velocities (*p* < 0.05); a reduction in RV free wall systolic strain (−21.1 ± 8.6 vs. −31.2 ± 11%, *p* < 0.001).
Ozyigit [34]	2010	Case–control study	21	32.3 ± 12.3	21	Right ventricular function by Doppler	DM1 patients showed a reduction in peak velocity (cm/s) of Sm (12.38 ± 2.91 versus 14.40 ± 2.25 *p* = 0.016), Em (11.91 ± 3.54 versus 14.39 ± 3.87 *p* = 0.037); Tei index was significantly higher in DM1 patients compared with controls (0.27 ± 0.17, *p* = 0.013).
**Left Ventricular Diastolic Dysfunction**							
Fayssoil [16]	2014	Case–control study	26	45.1 (10.9)	13	Diastolic function	Increased left atrium diameter and increased mitral deceleration time compared with healthy controls; no differences were found regarding mean peak E/A mitral ratio, mean peak lateral early diastolic velocity and mean peak septal early diastolic velocity.
Wahbi [26]	2011	Case–control study	39	37.5 ± 12.1	39	LV GLS	Speckle tracking GLS was able to identify LV contractility abnormalities in DM1 patients with normal LVEF. DM1 patients showed a lower apical 4 chambers GLS compared to controls (−17.8 ± 2.5 vs. −19.2 ± 2.3 *p* = 0.01), which significantly correlated with PR interval.
**Left atrial function**							
Bhakta [48]	2003	Based on a prospective multicenter registry	382	42.2 ± 12.3 (17.9–77.8)	-	Structural cardiac abnormalities	Structural cardiac abnormalities determined with cardiac imaging included left ventricular hypertrophy (19.8%), left ventricular dilatation (18.6%), left ventricular systolic dysfunction (14.0%), mitral valve prolapse (13.7%), regional wall motion abnormality (11.2%) and left atrial dilatation (6.3%).
Fragola [15]	1997	Prospective study	42	37 ± 12	41	Left Ventricular Diastolic Function	The most common abnormalities were increased deceleration time (>224 ms), prolonged isovolumic relaxation time (>103 ms) and reduced rate of decline of flow velocity in early diastole (<2.1 m/s^2^).

DM1: myotonic dystrophy 1; LVEF: left ventricular ejection fraction; LV GLS: left ventricular global longitudinal strain; LA: left atrial; RV: right ventricular; RVEF: right ventricular ejection fraction; (FAC: fractional area change).
